# Cases of Pregnancy with Mild Intellectual Disabilities Managed at a Japanese Perinatal Center

**DOI:** 10.31662/jmaj.2021-0005

**Published:** 2021-03-18

**Authors:** Shunji Suzuki

**Affiliations:** 1 Department of Obstetrics and Gynecology, Japanese Red Cross Katsushika Maternity Hospital, Tokyo, Japan

**Keywords:** mild intellectual disabilities, pregnancy, multidisciplinary collaboration, Japan

Intellectual disability (ID) is a generalized neurodevelopmental disorder characterized by impaired intellectual and adaptive functioning ^(1), (2), (3)^. Mild ID has been defined by an intelligence quotient (IQ) of 50-69 in addition to deficits in two or more adaptive behaviors that affect general living.

During pregnancy and childcare, women need to learn and practice a variety of new things; however, those with ID have difficulties in learning or practice. Thus, medical staffs have to care and support them in considering with the difficulties by multidisciplinary collaboration. In Japan, there have been various reports from the staff of local government agencies and nursing that summarized the care ^(4), (5), (6), (7), (8)^, but only a few reports from obstetricians.

Therefore, in this study, we reviewed the obstetric records to examine the clinical characteristics and perinatal outcomes of 12 Japanese pregnant women who had been issued the Rehabilitation Handbook (= certification) for mild ID and who delivered at our institute after 22 weeks of gestation between 2017 and 2020. The protocol for this study was approved by the Ethics Committee of the Japanese Red Cross Katsushika Maternity Hospital. Their comprehension in conversation was evaluated based on the impression of our trained midwives. In our institute, the Japanese version of Mother-to-Infant Bonding Scale (MIBS-J) ^(9)^ has been used to detect problems in a mother's feelings toward her baby at one month after delivery. In the MIBS-J, the mother is evaluated as having a mother-infant bonding if the score is 2 or less.

As shown in Table 1, the pregnant women with mild ID sometimes suffered from other mental disorders, including depression from various stresses in their lives. Because their partners also often have similar levels of ID and/or other mental disorders, a lot of social and medical support will be required in their pregnancies and/or childcare. They seemed to have enough love for their children; however, it was hard to make them feel the responsibility in and difficulty of childcare. After discharge, they all went home with their children, and there was appropriate childcare observed in them.

**Table 1.** Clinical Characteristics and Perinatal Outcomes of 12 Japanese Pregnant Women with Mild Intellectual Disabilities.

	Yes	
	Number	%
Total number	12	100
Maternal age		
<25 years	9	75
≥25 years	3	25
Multiparous woman	2	17
Psychiatric diseases with intellectual disabilities	5	42
Absence of partner	2	17
Partner having mental disorders	8 (/10)	80
Comprehension		
Easy reading, writing, and calculation	9	75
Easy understanding general conversation	2	17
Easy compliance of health guidance	1	8
Easy compliance of medical explanations	1	8
Serious fear against treatment and/or delivery	2	17
Image of postpartum life during pregnancy	8	67
Perinatal complications		
Hypertensive disorders	4	33
Impaired glucose tolerance	1	8
Fetal growth restriction	0	0
Obstetric outcomes		
Preterm delivery	2	17
Cesarean delivery	8	67
NICU admission	2	17
Healthy discharge of both mother and child	12	100
Maternal-child bonding at 1 month after delivery	11	92
Inappropriate childcare	0	0

NICU, neonatal intensive care unit.

We understand the small sample size of the current study; however, the incidence of hypertensive disorders during pregnancy in women with mild ID seemed to be high (33% vs. almost 10%: average incidence in Japan ^(10)^), which are supported by some recent reports ^(11), (12)^. The reasons for the increased hypertension among pregnant women with ID have been unclear; however, Brown et al. ^(11)^ suggested that there is a need to address modifiable risk factors for adverse outcomes among women with ID prior to and during pregnancy. On the basis of the current results, the fear against treatment and/or delivery in them may be associated with the development of sympathetic-stimulated hypertension. In any cases, a further large study is needed with consideration that wellness during pregnancy involves social, familial, and clinical support systems responsive to each woman's needs.

As expected, the pregnant women with mild ID had poor understanding of medical explanations; however, they were able to be brought up by patiently explaining and practicing in cooperation with local government agencies ^(1), (2), (3), (4)^. They thought that their children are cute and showed affection; however, they seemed to be poor at dealing with things that they were not interested in or difficult. In particular, breastfeeding at night was seemed to be difficult to learn based on our impression as previously reported ^(8)^. On the basis of the results of the MIBS-J, there was one mother with a possibility that she had problems regarding feelings for her baby; however, her mother-child bonding seemed to be formed with the child-rearing support by the public health nurses/midwives, although she seemed confused regarding her child-rearing life for one month after delivery. In this study, all mothers with mild ID could perform appropriate childcare with the support of those around them.

In addition, Nagai ^(8)^ was concerned that women with ID tend to refuse social supports during pregnancy and childcare. However, compared to those with other mental disorders such as schizophrenia spectrum disorders ^(13), (14)^, if they become aware of childcare difficulties as the result of time-consuming practice, their subsequent acceptance of social support will be smooth.

Although the subjects in this study were limited to women with mild ID, various supports seemed to be required and performed for them. However, one of the serious limitations of the examination may be that we examined only cases with mild ID. This is because those with moderate or higher ID (IQ of <50) often encounter inability in childcare less than 1 week after delivery ^(4), (8)^. Pregnant women with moderate or higher ID also seemed to love their children, although they might more likely experience maltreatment than those with no or mild disabilities ^(15)^; however, they usually cannot envision postpartum life with childcare, so medical staff have to fully evaluate and predict their childcare abilities by multidisciplinary collaboration ^(16)^. If their childcare abilities are not sufficient, support of the social care facilities such as infant care centers should be provided.

In conclusion, we reported cases of pregnancy with mild ID supported by multidisciplinary collaboration at a Japanese perinatal center.

## Article Information

### Conflicts of Interest

None

### Author Contributions

Shunji Suzuki: all project development, data management, data analysis, manuscript writing/editing.

### Approval by Institutional Review Board (IRB)

The study protocol was approved by the Ethics Committee of the Japanese Red Cross Katsushika Maternity Hospital (K2020-16).

### Informed Consent

Patients’ informed consent for publication of this report was obtained.
